# Electrophysiology of Heart Failure Using a Rabbit Model: From the Failing Myocyte to Ventricular Fibrillation

**DOI:** 10.1371/journal.pcbi.1004968

**Published:** 2016-06-23

**Authors:** Aditya V. S. Ponnaluri, Luigi E. Perotti, Michael Liu, Zhilin Qu, James N. Weiss, Daniel B. Ennis, William S. Klug, Alan Garfinkel

**Affiliations:** 1 Department of Mechanical and Aerospace Engineering, University of California Los Angeles, Los Angeles, California, United States of America; 2 Department of Radiological Sciences and Department of Bioengineering, University of California Los Angeles, Los Angeles, California, United States of America; 3 Department of Medicine - Division of Cardiology, University of California Los Angeles, Los Angeles, California, United States of America; 4 Department of Integrative Biology and Physiology, University of California Los Angeles, Los Angeles, California, United States of America; University of California San Diego, UNITED STATES

## Abstract

Heart failure is a leading cause of death, yet its underlying electrophysiological (EP) mechanisms are not well understood. In this study, we use a multiscale approach to analyze a model of heart failure and connect its results to features of the electrocardiogram (ECG). The heart failure model is derived by modifying a previously validated electrophysiology model for a healthy rabbit heart. Specifically, in accordance with the heart failure literature, we modified the cell EP by changing both membrane currents and calcium handling. At the tissue level, we modeled the increased gap junction lateralization and lower conduction velocity due to downregulation of Connexin 43. At the biventricular level, we reduced the apex-to-base and transmural gradients of action potential duration (APD). The failing cell model was first validated by reproducing the longer action potential, slower and lower calcium transient, and earlier alternans characteristic of heart failure EP. Subsequently, we compared the electrical wave propagation in one dimensional cables of healthy and failing cells. The validated cell model was then used to simulate the EP of heart failure in an anatomically accurate biventricular rabbit model. As pacing cycle length decreases, both the normal and failing heart develop T-wave alternans, but only the failing heart shows QRS alternans (although moderate) at rapid pacing. Moreover, T-wave alternans is significantly more pronounced in the failing heart. At rapid pacing, APD maps show areas of conduction block in the failing heart. Finally, accelerated pacing initiated wave reentry and breakup in the failing heart. Further, the onset of VF was not observed with an upregulation of SERCA, a potential drug therapy, using the same protocol. The changes introduced at the cell and tissue level have increased the failing heart’s susceptibility to dynamic instabilities and arrhythmias under rapid pacing. However, the observed increase in arrhythmogenic potential is not due to a steepening of the restitution curve (not present in our model), but rather to a novel blocking mechanism.

## Author Summary

Ventricular fibrillation (VF) is one of the leading causes of sudden death. During VF, the electrical wave of activation in the heart breaks up chaotically. Consequently, the heart is unable to contract synchronously and pump blood to the rest of the body. In our work we formulate and validate a model of heart failure (HF) that allows us to evaluate the arrhythmogenic potential of individual and combined electrophysiological changes. In diagnostic cardiology, the electrocardiogram (ECG) is one of the most commonly used tools for detecting abnormalities in the heart electrophysiology. One of our goals is to use our numerical model to link changes at the cellular and tissue level in a failing heart to a numerically computed ECG. This allows us to characterize the precursor to and the risk of VF. In order to understand the mechanisms underlying VF in HF, we design a test that simulates a HF patient performing physical exercise. We show that under fast heart rates with changes in pacing, HF patients are more prone to VF due to a new conduction blocking mechanism. In the long term, our mathematical model is suitable for investigating the effect of drug therapies in HF.

## Introduction

Heart failure is the leading cause of death and one of the most common causes of hospitalization in the United States. However, the mechanisms that lead to heart failure are still poorly understood. Evaluating the underlying cardiac electrophysiology (EP) can help in the treatment of cardiac arrhythmias and other consequences of heart failure. In this regard, computational biventricular models enable us to investigate the effect of changes in EP parameters and, by comparing the results to empirical clinical evidence, to refine the mechanisms of heart failure.

The definition of heart failure encompasses a broad range of conditions each with a compromised cardiac function. Consequently there is not a “true” single model of heart failure, but rather a range of subclasses, each requiring a separate model. Here, to narrow our scope and enable the formulation of a computational model, we focus on congestive heart failure (CHF). Although the EP of patients with congestive heart failure is still not uniquely defined, there are several common features reported in the literature [1]. For example, one of the defining characteristics of CHF is a prolonged action potential duration (APD) in myocytes [2]. This suggests abnormalities in repolarization currents, specifically in the voltage gated potassium channels. Moreover, the calcium transient is longer in duration and lower in amplitude [3]. The prolonged APD and longer and lower calcium transient suggest a potential mechanism in which the heart is compensating for reduced cardiac output by increasing the time of contraction. Under normal heart rates, these changes might not have a drastic effect on the propagation of the electrical wave of activation. However, during elevated heart rates, abnormalities arise in the voltage dynamics. Specifically, when compared to the normal myocyte, action potential (AP) and calcium alternans occur at a longer pacing cycle length in the failing myocyte. At the tissue level, alternans may be spatially concordant, that is all the myocytes alternate longer and shorter action potentials, or they may be spatially discordant if myocytes in different regions show opposite responses. For example, Qu et al. [4] have shown that by pacing a square block of tissue at 200ms concordant alternans arises, whereas spatially discordant alternans is present at pacing cycle length equal to 180ms, when myocytes have a long AP in one region and a short AP in another during the same excitation cycle. In the subsequent cycle, the opposite happens, that is, regions with previously long AP show short AP and vice versa. Together with changes in the myocyte EP, hearts suffering from congestive heart failure show a lower and more isotropic conduction velocity with respect to a healthy heart. In a normal myocyte, a high density of gap junctions is found at its end, while in a failing cell remodeling causes myocytes to revert to a juvenile state where gap junctions move to the crossfiber and sheet normal directions [5]. Due to this remodeling, the likelihood of a cell exciting a neighboring cell aligned in the fiber direction has decreased, and consequently the electrical conduction velocity is reduced [1].

Modeling cardiac tissue EP requires the combination of two underlying physics: (1) cell level ion channel mediated currents that can be described as the solution to a set of ordinary differential equations (ODEs); and (2) cell-to-cell diffusion via gap junctions as the solution to a reaction-diffusion partial differential equation (PDE) [6]. In this regard, our group has previously developed, verified, and validated a multiscale model [7] to simulate the EP of a healthy heart. Specifically we have reproduced the correct activation, electrocardiogram (ECG), and wave dynamics in a healthy rabbit heart, including the generation of ventricular fibrillation by an ectopic beat. In the present study, we modified the model to capture the known characteristic features of a failing myocyte and analyze their effect on ventricular EP.

Other groups have investigated numerically the effect of failing cell electrophysiology and structural remodeling on the heart’s susceptibility to ventricular fibrillation. For example, Gomez et al. [8] have investigated the effect of fibrosis and cellular uncoupling on the safety factor for conduction. In heart failing conditions, this safety factor is reduced in one-dimensional simulations. In a subsequent study, Gomez et al. [9] have also shown how intermediate levels of fibrosis and cellular uncoupling lead to wave reentry in 2D simulations. Other studies have focused on understanding the link between changes in ion channels expression and biomarkers of the action potential and calcium transient. For example, Walmsley et al. [10] investigate the mRNA expression in healthy and failing myocytes to predict the electrophysiology remodeling in heart failure. In doing so, Walmsley et al. consider that population variability is a key factor in constructing robust computational models. Intersubject variability is particularly important when the in silico model is adopted to test drug therapies. In silico testing of drug therapies is indeed one of the goals of current EP models (see, e.g., [11]).

Using our model we aim to investigate the mechanisms leading to ventricular fibrillation (VF) in heart failure and investigate which clinical signs (e.g., features in the ECG) are precursors to VF. Moreover, we isolate myocyte-level-changes responsible for increased arrhythmogenesis. This computational model allows us to determine if both membrane and calcium changes are necessary to generate VF in the failing heart, and if changes in conduction velocity and anisotropy (due to gap junction remodeling) are also key factors. In order to achieve these goals, we focused on the verification and validation of our model from the cellular to the biventricular level, with particular attention to the electrocardiogram in the normal and failing conditions.

## Models

In order to simulate the electrophysiology of heart failure, we need to develop and validate a single cell failing EP model and the numerical methods necessary to simulate the failing EP in a biventricular model. We present both the EP model and the numerical methods in the following.

The heart failure literature reviews by Nattel et al. [1] and Gomez et al. [12] provide an extensive summary of the changes observed in HF. In addition to the ion channel remodeling, altered calcium handling, and gap junction remodeling, structural changes (i.e., repolarization heterogeneities and fibrosis) play a major role in HF [12, 13]. Indeed, arrhythmia is initiated by the prolonged APD and longer calcium transient but is sustained by structural changes.

### Electrophysiology model of the failing myocyte

Our first aim is to formulate a cell model that: 1) reflects the ion channel modifications observed in heart failure; and 2) reproduces the characteristic action potential, calcium transient, sodium transient, and restitution curve observed in failing myocytes. We subdivide the myocyte EP changes depending on their direct effect on membrane ion channels or calcium dynamics.

#### Membrane current changes

Although there is not a single cell model of heart failure, and significant variations are possible in the heart failure population, the following changes in ionic currents are common across different studies reported in the literature:

- Downregulation of the peak slow *g*_to,s_ and fast *g*_to,f_ potassium outward conductances [14, 15].
- Downregulation of the peak potassium delayed rectifier conductance *g*_K_*s*__[15–17].
- Downregulation of the peak potassium inward rectifier conductance *g*_K_1__[14, 15].
- Upregulation of the strength of the sodium calcium exchange conductance *g*_NaCa_[14, 18, 19].
- Presence of a persistent leak sodium current. Among others, as reviewed by Noble et al. [20], congestive heart failure myocytes exhibit elevated intracellular sodium concentrations. Despa et al. [21] also show that a sodium influx is present even with sodium channel blockers. They suggest that this influx could be attributed to the presence of background sodium leak channels. According to these findings, we do not directly change *g*_*Na*_ in our model, but introduce a leak current by changing the *m*, *h*, *j* gate kinetics of the sodium channel in a manner that is mathematically equivalent to a persistent background Na leak resulting in an elevated sodium concentration. As originally described by Luo and Rudy, the fast sodium current is defined as
  $$I_{\text{Na}}=g_{\text{Na}}m^{3}hj\left(V-E_{\text{Na}}\right)$$
  where *g*_Na_ is the peak sodium conductance, *E*_Na_ is the Nernst potential, and *m*, *h*, *j* are the variables which control, respectively, the activation gating, the fast inactivation gating, and the slow inactivation gating. We introduce a persistent leak current by setting the *m*, *h*, and *j* variables to range between 0.01 and 1, such that *I*_*Na*_ is always greater than zero (in the normal cell model *m*, *h*, and *j* range from 0 to 1).

The membrane current changes are summarized in Table 1.

**Table 1:** Membrane current changes from normal to failing cell model.

Variable	Normal	Failing
*g*_to,s_ [mS/*μ*F]	0.04	0.026
*g*_to,f_ [mS/*μ*F]	see Table 3	
*g*_K_*s*__ [mS/*μ*F]	see Table 3	
*g*_K_1__ [mS/*μ*F]	0.3	0.15
*g*_NaCa_ [*μ*M/s]	0.84	1.68

#### Calcium dynamics changes

The changes in calcium dynamics are specific to the EP cell model employed in the simulations. We use the Mahajan et al. [22] cell model that has been formulated to accurately describe the calcium dynamics during rapid pacing. This is particularly important in the current study that aims at investigating possible mechanisms of ventricular fibrillation induced by fast heart rates in heart failure. We introduce the following changes in the calcium dynamics of the failing cell model with respect to the normal cell model:

- Lower strength of uptake *v*_up_.
- Higher release slope *u*.
- Longer submembrane-myoplasm diffusion time constant *τ*_d_.
- Longer non-junctional sarcoplasmic reticulum (NSR) to junctional sarcoplasmic reticulum (JSR) relaxation time *τ*_a_. As highlighted in [22], *τ*_a_ plays a key role in determining the onset of calcium driven alternans.
- Lower threshold for steep release function *c*_sr_.

The calcium dynamic changes are summarized in Table 2.

**Table 2:** Calcium handling changes from normal to failing cell model.

Variable	Normal	Failing
*v*_up_ [*μ*M/ms]	0.4	0.27
*u* [mS^−1^]	11.3	22.5
*τ*_d_ [mS]	4	6
*τ*_a_ [mS]	100	200
*c*_sr_ [*μ*M/l]	90	50

#### Changes in conduction velocity and APD gradients

In addition to membrane and calcium handling changes, other changes occur in heart failure. Due to downregulation of Connexin43 (Cx43), the myocytes’ gap junctions undergo remodeling that leads to increased lateralization (decreased anisotropy) [1] and slower conduction velocity [23–26]. In the normal cell model the diffusion along the fastest (fiber), medium (cross fiber), and slower (normal to the fiber sheet) directions scales according to a 4:2:1 ratio [27]. In the failing model we modify the ratio of diffusivities to be 2:1:1 (see section titled “Full Heart Model Construction”).

In the ECG, a morphologically correct T-wave is due to a spatial repolarization sequence in the myocardium. This repolarization sequence is produced by a spatial distribution of APDs, which are due to apex-to-base and transmural gradients for the peak fast potassium outward conductance *g*_to,f_ and the peak potassium delayed rectifier conductance *g*_K_*s*__ (See [7] and references therein). In order to assign different *g*_to,f_ and *g*_K_*s*__ values throughout the myocardium, we divide the biventricular geometry into nine distinct regions [28] in the transmural (epicardium, “M”, and endocardium) and apex-to-base (apex, mid, base) directions. In the current work, we modify the values reported in [7] to account for reduced APD gradients throughout the ventricles, especially in the transmural direction, during heart failure [29]. The resulting changes to *g*_to,f_ and *g*_K_*s*__ are reported in Table 3. Elshrif et al. [30] have compiled a list of remodeled currents in heart failure. The findings in Table II of their work shows that the largest differences transmurally involved, as the changes made in our work, the *I*_*to*_ and *I*_*Ks*_ currents.

**Table 3:** Transmural and apex-to-base APD gradients in the normal and failing cell models.

Cell type	Normal			Failing		
Cell position	*g*_tof_ [mS/*μ*F]	*g*_ks_ [mS/*μ*F]	APD [ms]	*g*_tof_ [mS/*μ*F]	*g*_ks_ [mS/*μ*F]	APD [ms]
Epi—Apex	0.110	0.263	158	0.080	0.125	220
Epi—Mid	0.110	0.194	168	0.080	0.108	227
Epi—Base	0.110	0.139	179	0.080	0.091	235
M—Apex	0.110	0.103	189	0.080	0.083	240
M—Mid	0.110	0.072	202	0.080	0.075	246
M—Base	0.110	0.049	217	0.080	0.067	252
Endo—Apex	0.094	0.136	182	0.062	0.091	244
Endo—Mid	0.094	0.097	195	0.062	0.083	250
Endo—Base	0.094	0.069	208	0.062	0.075	256

#### Purkinje cell remodeling

In addition to myocardial cells, Purkinje cells (further discussed in the “Full Heart Model Construction” section) also undergo remodeling during heart failure [31]. However, in contrast with myocardial cells, the action potential of Purkinje cells is not prolonged as illustrated in Fig. 7E and F of Han et al. [31]. Therefore, the activation of the myocardium through the Purkinje and the possibility of reentry in the Purkinje network during VF would not be significantly affected in our model. For these reasons and because here we focus on the effect of failing myocardial cells EP, we do not include changes in Purkinje cell EP during heart failure. We remark that a failing Purkinje cell model should be included if dynamic instabilities in the Purkinje network are considered.

### Numerical methods

In the following we describe the pacing protocols used at the single cell level, the simulation procedure at the 1D cable level, and the construction of the model for biventricular simulations. Due to the lowered conduction velocity in heart failure, we also perform benchmark tests to ensure accuracy of the propagation of the voltage wave of activation with respect to mesh size.

#### Single cell pacing protocol

In single cell simulations, 1D cell cables, and full biventricular EP simulations, we begin by prepacing the single normal and failing cell models one thousand times. This prepacing is carried out at the same cycle length as the simulation for which the cell is being used. Further, this ensures that each cell has reached steady state conditions, i.e., the difference between state variables in subsequent beats is minimal. Using this prepacing protocol, our simulations are representative of a typical heart beat at a specific cycle length. Each cycle in the prepacing protocol is initiated with a stimulus current applied for 2ms.

In order to validate the single cell model, following the prepacing protocol, we record the action potential, the calcium transient, the intracellular sodium, and we compute the restitution curve. We compared these results at the single cell level with the features expected in the literature for the failing myocyte (see section titled “Failing versus normal myocyte models”).

#### Restitution pacing protocol

APD restitution curves are generated for pacing cycle lengths (PCL) between 400ms and 200ms using the following protocol adapted from [22]:

1. The myocyte is paced according to the protocol described in the previous section at PCL = 400ms.
2. At each decreasing PCL, the myocyte is paced an additional twenty times until steady state is reached and the differences in state variables at the end of every subsequent beat is minimal.
3. Voltage traces are recorded during the last two beats at each PCL, and APD_90_ and diastolic intervals (DI = PCL−APD_90_) are computed and recorded.
4. PCL is reduced by 5ms and steps 2–3 are repeated until PCL = 200ms.

APD restitution curves are plotted as APD_90_ versus diastolic interval (DI) whereas dynamic restitution curves are plotted as APD_90_ versus PCL.

#### Cable model pacing protocol

After completing EP simulations at the single cell level, we ran simulations with homogeneous and heterogeneous cables. Each cable is made of 300 cells, each 0.02*cm* long. Every cell in the homogeneous cable represents one of the nine transmural or apex-to-base regions in the full heart. In contrast, the heterogeneous cable is made of three different cell types, each occupying a third of the cable and representing the transmural or apex-to-base EP gradients. For example, an apex-to-base heterogeneous cable consists of 100 apical cells, 100 center cells, and 100 basal cells.

The electrical propagation in the cable is governed by the monodomain equation of electrophysiology

$$\chi \left(C_{m}\frac{\partial V}{\partial t}+I_{\text{ion}}\left(u\right)\right)-\nabla \cdot \left(\sigma \nabla V\right)=I_{\text{stim}}\;,$$ (1)

where *V* is the transmembrane voltage, *χ* is the surface area to volume ratio of a cell, *C*_*m*_ is the capacitance of a unit area of cell membrane, *σ* is the conductivity tensor and $I_{\text{stim}}$ is the applied stimulus current. The conductivity tensor can be expressed in terms of the diffusion tensor **D** as *σ* = *χC*_m_ **D**. In the case of cable simulations, only the primary direction’s diffusion value is modified (D_normal_ = 0.001cm^2^/ms and D_fail_ = 0.0005cm^2^/ms, written as scalars for 1D diffusion). Note that Li et al. [32] have shown no statistically significant differences between cell capacitance in normal and failing ventricular myocytes and thus this value was not altered in our model. The ionic current $I_{\text{ion}}\left(u\right)$ is a function of the cell state variables **u**, which are governed by a set of ordinary differential equations (ODEs) describing the cell electrophysiology. In this work, the state variables **u** obey the Mahajan et al. [22] cell model and the governing system of ODEs is solved using Euler’s method with adaptive time step. $I_{\text{ion}}$ is coupled to the monodomain equation using ionic current interpolation, reaction-diffusion operator splitting with adaptive time stepping [33], and the C-LL lumping scheme as described in Krishnamoorthi et al. [34].

A stimulus is applied to one end of the cable for 5 ms while a no-flux boundary condition is imposed at the opposite end. Note that a longer stimulus is required in the cable simulations than in the single cell analyses to overcome source-sink mismatch. Cell EP measurements are made at the middle of each cable region such that edge effects are minimal.

We can examine the presence of voltage alternans using space versus time voltage plots. In these plots, concordant alternans will manifest itself as beat-to-beat alternation in APD, whereas discordant alternans will manifest itself as spatial variation in APD in the same beat.

#### Full heart model construction

Following the validation of the cell model using single cell and cable simulations, we used the finite element method to solve the monodomain equations of electrophysiology in a full biventricular model. The model geometry and microstructure were constructed from diffusion tensor magnetic resonance imaging (DTMRI) of a New Zealand white rabbit heart. The recommendations of the Institutional Animal Care and Use Committee at the University of California, Los Angeles (UCLA) and the National Institutes of Health Guide for the Care and Use of Laboratory Animals were followed during animal handling and care. Animal protocol #2008-161-12 was approved by the UCLA Chancellor’s Animal Research Committee.

**Fig 1:**
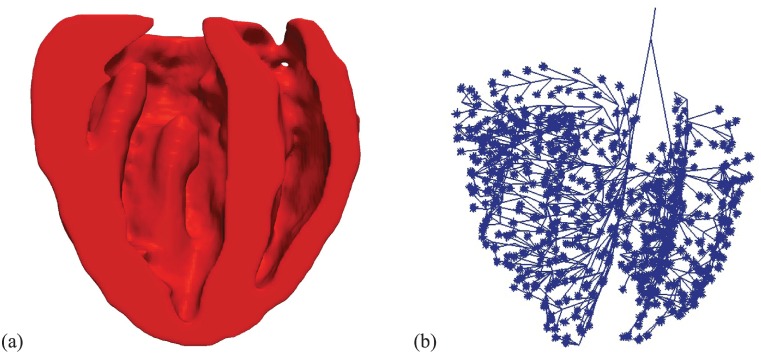
(a) Longitudinal section of the biventricular heart geometry; (b) Purkinje structure with terminal Purkinje muscle junctions.

The biventricular heart geometry (Fig 1a) was meshed with ≈ 830k trilinear hexahedral elements with edge length *h* = 200*μ*m, corresponding to ≈ 900k nodes. The model also includes a detailed Purkinje structure with ≈ 500 Purkinje muscle junctions (Fig 1b). A detailed description of the construction of the finite element mesh and fiber interpolation scheme is discussed in [7].

To model the increased lateralization of gap junctions due to downregulation of Connexin 43 (Cx43), the diffusion constants in **D** are decreased in the fiber and cross-fiber directions, but not in the sheet-normal direction, where the normal diffusion value is maintained:

$$\text{diag}\left(D_{\text{normal}}\right)=\left[\begin{array}{ccc} 0.001 & 0.0005 & 0.00025 \end{array}\right]{\text{cm}}^{2}/\text{ms}\;;$$ (2)

$$\text{diag}\left(D_{\text{fail}}\right)=\left[\begin{array}{ccc} 0.0005 & 0.00025 & 0.00025 \end{array}\right]{\text{cm}}^{2}/\text{ms}\;.$$ (3)

Normal diffusion values in the fiber and cross fiber directions were chosen according to [35, 36], while in the failing heart diffusion was reduced by half in the fiber direction according to [37].

The myocardium is activated through the Purkinje system, which we model using a tree of 1D cable elements. A stimulus current of 50,000 *μ*A/cm^3^ is applied to the terminal node of the Purkinje tree representative of the atrio-ventricular node. The ionic changes in the Purkinje elements are governed by the Corrias et al. cell model [38] with the following diffusion coefficients

$$\text{diag}\left(D_{\text{Purkinje}}\right)=\left[\begin{array}{ccc} 0.0032 & 0.0032 & 0.0032 \end{array}\right]{\text{cm}}^{2}/\text{ms}.$$

In order to overcome the effect of source-sink mismatch, the 1D-Purkinje cable elements are coupled to the 3D-myocardial elements through the Purkinje muscle junctions (PMJ) and the transfer of current follows Kirchhoff’s law. A description of the required density of PMJs and the construction of the Purkinje model to produce the correct activation sequence are reported in [7].

As presented in [7], an important validation criteria and output computed with an EP numerical model is the electrocardiogram. In our simulation, the ECG was computed using the following equation [39]:

$$\text{ECG}\left(t\right)=\int_{\Omega }\nabla V\left(x,t\right)\cdot \left(D\left(x\right)\cdot \nabla \left(\frac{1}{R(x)}\right)\right)\;d\Omega ,$$ (4)

where *R*(**x**) is the distance between the ECG lead and **x**, a point in the myocardium domain Ω. The location of the six leads is shown in Fig. 5A of [7].

The in-house C++ finite element code, biventricular geometry, microstructure data, and cell models are available at https://github.com/wsklug/UCLA_CMG.

#### Benchmark study for mesh convergence

**Fig 2:**
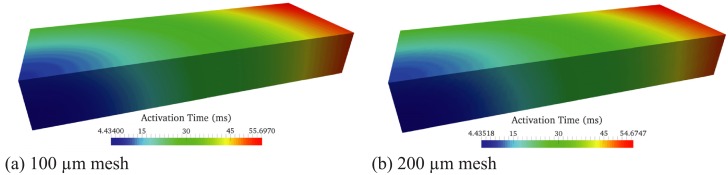
Activation times in a rectangular volume mesh with initial stimulus placed at the bottom left corner for (a) 100 *μ*m and (b) 200 *μ*m mesh sizes. Results obtained using a mesh with edge length equal to 200 *μ*m are converged within 2% error to the results obtained with a finer 100 *μ*m edge length mesh.

Mesh convergence analyses performed with normal conduction velocity have determined that a maximum element edge length of 200*μ*m is required to achieve correct myocardial activation times with no artifactual wavebreak. These analyses were performed on rectangular geometries (3mm × 7mm × 20mm) with a cube of activation (edge length of 1.5mm) at the bottom left corner, as described by Niederer et al. [40] (see Fig. 1 in [40]). In order to ensure that a maximum element edge length of 200*μ*m is still appropriate to model wave propagation in the current studies, we reran the convergence analyses for the lower conduction velocity value characteristic of heart failure (Fig 2). The results of the benchmark problem are shown in Fig 2. The activation times at the upper right corner opposite to the stimulus site for the 100*μ*m and 200*μ*m meshes are, respectively, 55.7ms and 54.7ms. The activation delay in the 200*μ*m mesh is less than 2% of the activation time computed with the 100*μ*m mesh.

## Results

Using the models and simulation protocols described earlier, we proceed to validate our single myocyte model, analyze the EP of 1D cables of failing versus healthy myocytes, and investigate VF mechanisms in full biventricular models based on the validated failing myocyte.

### Failing versus normal myocyte models

**Fig 3:**
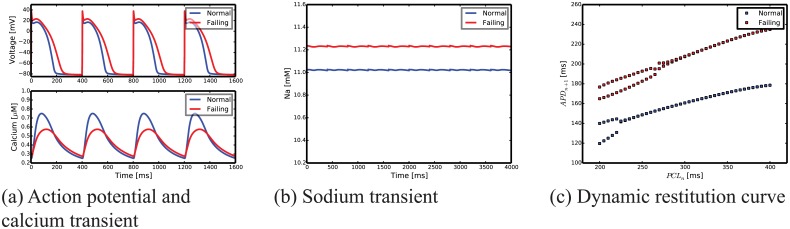
Comparison between normal and failing basal-epicardial myocyte models showing the characteristic EP of a failing myocyte: (a) longer action potential (as seen in Fig. 1 of [2]) (top); lower, slower, and longer calcium transient (as seen in Fig. 1 of [3]) (bottom); (b) elevated intracellular sodium; and (c) early onset of alternans.

By modifying the ion channels as described in the section titled “EP model of the failing myocyte”, we are able to reproduce the characteristic EP of a failing myocyte [1, 12, 19, 30]. Specifically, when compared to a normal myocyte, our failing cell model shows:

1. A longer action potential (Fig 3a top).
2. A lower, slower, and longer calcium transient (Fig 3a bottom).
3. An elevated intracellular sodium concentration (Fig 3b).
4. An early onset of alternans as apparent in the dynamic restitution curve (Fig 3c).

In Fig 3 we compare the electrophysiology of a normal and failing basal-epicardial myocyte. The same comparison is carried out in the supporting material for all nine cell types included in our biventricular model to simulate the apex-to-base and transmural APD gradients (Table 3). All transmural and apex-to-base cell regions in our model show the same characteristic differences listed above between healthy and failing myocytes (see supporting material.)

As discussed previously, due to both intersubject variability and the broad definition of congestive heart failure, there is not a single parameter set describing the characteristics of a failing myocyte. In order to assess the robustness of the chosen parameters regarding the single cell action potential and calcium transient, we perform preliminary uncertainty quantification (UQ) analyses. In these analyses, we perturbed each of the parameters governing the heart failure cell model by ±10% using a random uniform distribution, prepace the single cells in the nine transmural and apex-to-base regions, and record their states for one beat. We then plot the upper and lower bounds for the action potential and calcium transients in the nine cell regions.

The action potential UQ plots show that a ±10% variation in parameter values leads to an APD90 difference of approximately ±7% (see supporting material). Moreover, the lower bound APD for the failing cell resulting from the UQ analyses in any of the nine regions is higher than the corresponding APD for a normal myocyte. This is significant because, as discussed in the following sections, the onset of VF will rely on the existence of longer APD regions and corresponding functionally refractory tissue.

The upper and lower bounds for the Ca concentration computed in the UQ analyses show that ±10% cell parameter variation maintain the slower, lower, and longer calcium transient with respect to the normal myocyte (see supporting material). This persistent slow calcium recovery is also important in initiating wave propagation instabilities since it leads to calcium driven alternans.

In summary, preliminary UQ single cell analyses show that a modest variation in cell parameters does not alter the key features in cell electrophysiology that play an important role in the onset and propagation of wave instability and VF.

### One dimensional cable simulations at normal and rapid pacing

**Fig 4:**
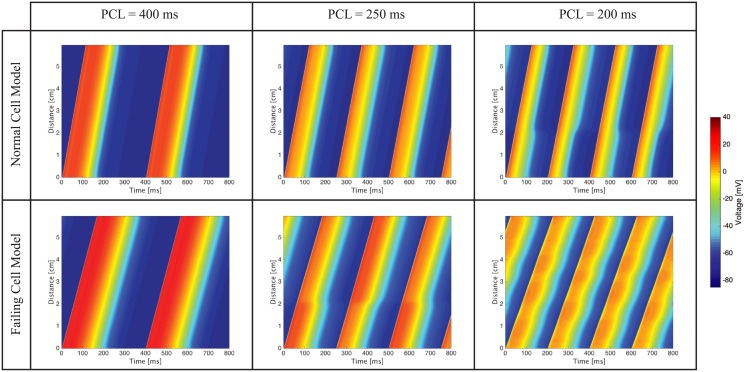
Homogeneous cable simulations showing increased activation times in the failing cell cables. Discordant alternans is visible at PCL = 250ms and PCL = 200ms in the failing cell cable. In contrast, in the normal cell cable, moderate concordant alternans is visible at PCL = 250ms and discordant alternans appears at PCL = 200ms.

Wave propagation in homogeneous cell cables at PCL of 400ms reveals increased activation times in the simulations performed with the failing cell model (Fig 4) with respect to the simulations performed with the normal cell model. This activation delay is largely due to a decreased diffusion coefficient in the failing cell cable. No alternans is visible at this resting PCL. At a faster PCL equal to 250ms, the normal cable shows concordant alternans, which is visible due to the alternating shades of dark blue corresponding to the resting state. In contrast, discordant alternans is present in the failing cell cable at PCL equal to 250ms. For example, at locations within 2cm from the starting edge of the cable, we notice, in subsequent beats, a long APD followed by a short APD and then a long APD. This pattern switches at a location more distant than 2cm from the cable edge. Finally, a PCL equal to 200ms produces discordant alternans also in the normal cell cable and accentuates the discordant alternans evident in the failing cell cable.

**Fig 5:**
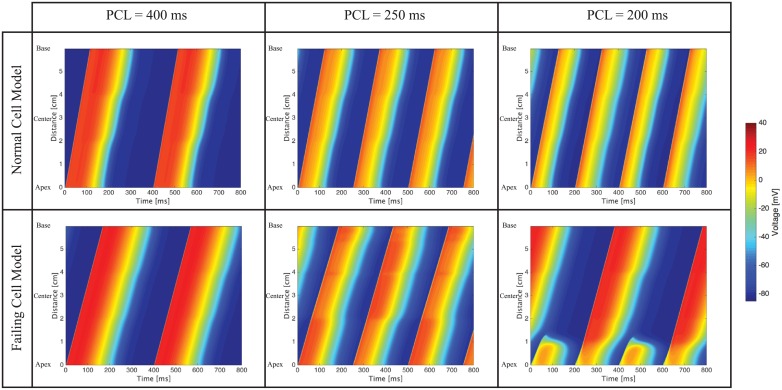
Apex-to-Base cable simulations. At PCL = 400ms, the APD gradient is apparent, especially in the normal cell cable. At PCL = 250ms no alternans is visible in the normal cell cable whereas discordant alternans is visible in the failing cell cable. At PCL = 200ms, the normal cell cable shows concordant alternans whereas the failing cell cable presents complete conduction block.

**Fig 6:**
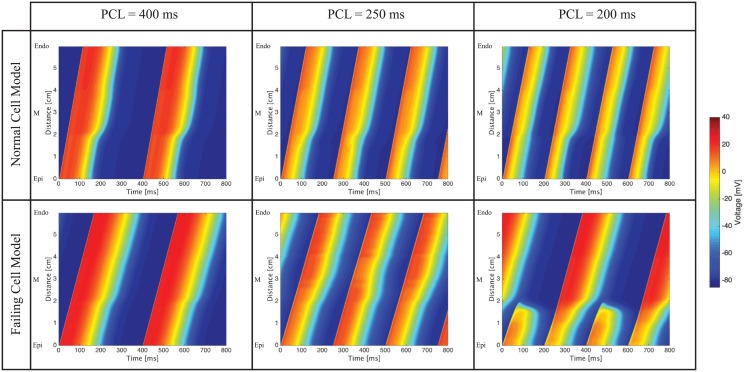
Transmural cable simulations. At PCL = 400ms, the APD gradient is apparent, especially in the normal cell cable. At PCL = 250ms a slight concordant alternans is visible in the normal cell cable, whereas discordant alternans is visible in the failing cell cable. At PCL = 200ms, the normal cell cable shows discordant alternans whereas the failing cell cable presents complete conduction block.

The apex-to-base and transmural cable simulations produce similar results and therefore we address together the common features observed. The cable heterogeneity is visible at normal pacing conditions (PCL = 400 ms): as the wave progresses through the cable, the APD changes because of the different cell types (e.g., Fig 5). At PCL equal to 250ms, the apex-to-base normal cable shows regular activation in subsequent beats whereas the transmural cable has slight concordant alternans at the boundary between the epicardial and M cell. Similar to the homogeneous cable simulations, both the failing apex-to-base (Fig 5) and transmural cables (Fig 6) show discordant alternans at PCL of 250ms. Finally, at PCL of 200ms the normal apex-to-base and transmural cables exhibit, respectively, concordant and discordant alternans, whereas the failing cables show 2:1 complete conduction block.

### Normal versus failing biventricular heart model

**Fig 7:**
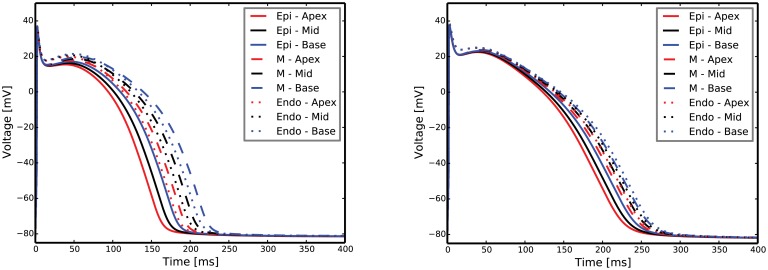
Normal (left) and failing (right) transmural and apex-to-base action potentials. In the failing heart, we notice the longer action potential and the reduced transmural and apex-to-base gradients.

By implementing the gradients in *g*_*to*,*f*_ (peak fast potassium outward conductance) and *g*_*Ks*_ (peak potassium delayed rectifier conductance) reported in Table 3 into the biventricular model, we obtain the transmural and apex-to-base APD gradients characteristic of a failing heart, i.e. reduced APD gradients—especially in the transmural direction—when compared to normal heart gradients (Fig 7).

**Fig 8:**
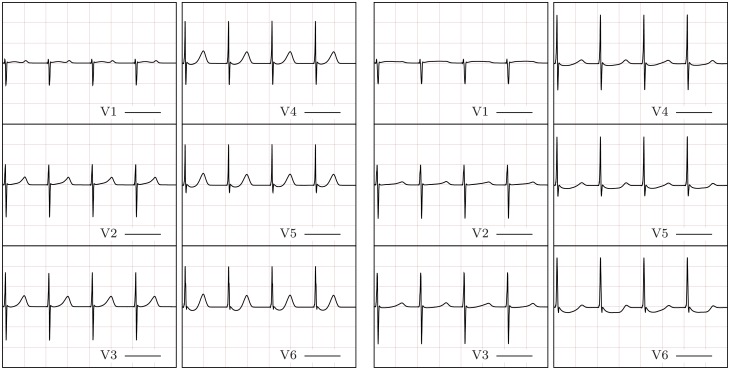
Normal (left) and failing (right) ECG at rest (PCL = 400ms). The failing heart ECG shows slight widening of QRS waves, lower T-wave peaks in all leads, and marked ST-segment depression in leads V5 and V6.

Using the cell model and the biventricular finite element model described earlier, we proceed to stimulate the normal and failing hearts at PCL = 400ms for four beats and compute the corresponding ECG (Fig 8). At this PCL, overall normal QRS waves and QRS wave progression are visible in the ECG obtained for both the normal and the failing hearts. Moreover no fractionations or slurring are present in either the normal or the failing heart ECG. However, the QRS waves in the failing heart ECG are slightly wider than in the normal heart ECG and marked differences between the normal and the failing hearts are present regarding the T-wave. Specifically, the T-wave peaks are lower in all leads and ST-segment depression is present in leads V5 and V6 for the failing heart.

#### T wave and QRS alternans

**Fig 9:**
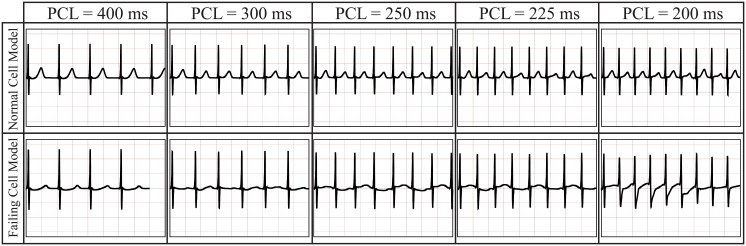
ECG traces for representative lead V5 at different PCLs for both the normal and the failing heart models. From left to right: PCL = 400ms, PCL = 300ms, PCL = 250ms, PCL = 225ms, and PCL = 200ms. From top to bottom: normal and failing heart models. In the normal biventricular model, T-wave alternans increases from PCL≈ 250 to PCL = 200ms. However, no T-wave inversion occurs in the normal biventricular model. In contrast, T-wave alternans appears at PCL = 300ms in the failing biventricular model and progresses to include T-wave inversion and irregular T-waves at faster PCLs.

At resting PCL, as stated above, the main differences between normal and failing heart ECGs are in T-wave morphology and slight widening of the QRS. In order to investigate the effect of a faster PCL on the failing heart, we repeat the biventricular heart simulations at PCL = 300ms, PCL = 250ms, PCL = 225ms, and PCL = 200ms. With the aim of comparing the ECGs obtained at each PCL for both the failing and the normal biventricular models, we report, as representative, the ECG traces obtained at lead V5 (Fig 9). The full ECGs for all cases are reported in the supporting material for additional reference. In the normal biventricular model, a shorter (faster) PCL initiates moderate T-wave alternans, that is, the amplitude and rising slope of the T-wave alternate in subsequent beats. T-wave alternans gradually appears at PCL≈ 250ms and becomes more marked at PCL = 200ms. However, no T-wave inversion is present in the normal heart at any PCL≥ 200ms. T-wave alternans appears earlier (PCL = 300ms) and more markedly in the failing heart model. At PCL = 250ms and PCL = 225ms, alternate T-waves are inverted while at PCL = 200ms, the T-waves become highly irregular. In contrast to the normal heart ECG, the failing heart ECG also presents moderate QRS alternans at rapid PCL, i.e., PCL = 225ms and PCL = 200ms.

#### Delta APD maps

**Fig 10:**
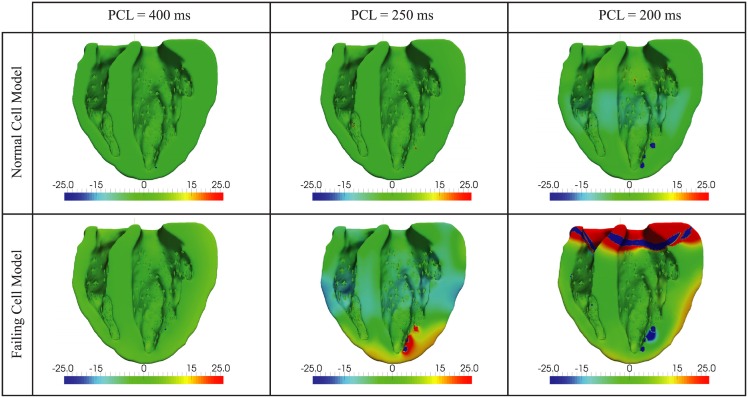
Delta APD maps generated by computing the difference between the APDs of two subsequent beats measured in milliseconds. No alternans is evident at PCL = 400ms. At PCL = 250ms, discordant alternans is present in both the normal and the failing biventricular model, although the alternans is very slight in the normal model. At PCL = 200ms, a more marked discordant alternans is evident in the normal biventricular model, whereas full conduction block appears in the basal region of the failing model.

The APD maps shown in Fig 10 are computed by taking the difference in APDs from two consecutive beats (after several beat of prepacing). At rest with PCL = 400ms, there is no difference in the delta APDs in the simulations obtained with the normal and failing cell models. At faster pacing with PCL = 250ms, the model based on the normal myocyte EP exhibits minor (< 5 ms) spatially discordant alternans whereas this phenomenon is more pronounced in the model based on the failing cell EP.

In Fig 10, we also observe that conduction block develops near a few apical PMJs sites at PCL = 250ms and PCL = 200ms. This occurs because the activation wave fails to propagate at a terminal Purkinje junction due to rapid pacing. Following a stimulus, Purkinje cells [38] require a longer recovery time than myocardial cells. Indeed, in 1D cable simulations we observed that a higher stimulus current was required to sustain action potentials during rapid pacing. In our simulation, the stimulus current through the Purkinje network was not a function of PCL and this results in blocking at a distal branch site. The majority of the PMJs attached to this final Purkinje branch remain electrically silent throughout the remainder of the beat but some PMJs undergo retrograde activation from the surrounding myocardial cells (see also figure provided as supplementary material.)

Due to the lengthening of the APD in the failing cell model, we also observe that conduction block develops near a few PMJs sites at PCL = 250ms. This also leads to retrograde activation from the myocardium into the Purkinje network near those nodes. Finally, at PCL = 200ms, discordant alternans is more readily visible in the simulations using a normal cell model whereas there is a complete conduction block near the base of the heart containing the failing cell model.

#### Rapid ventricular stimuli

**Fig 11:**
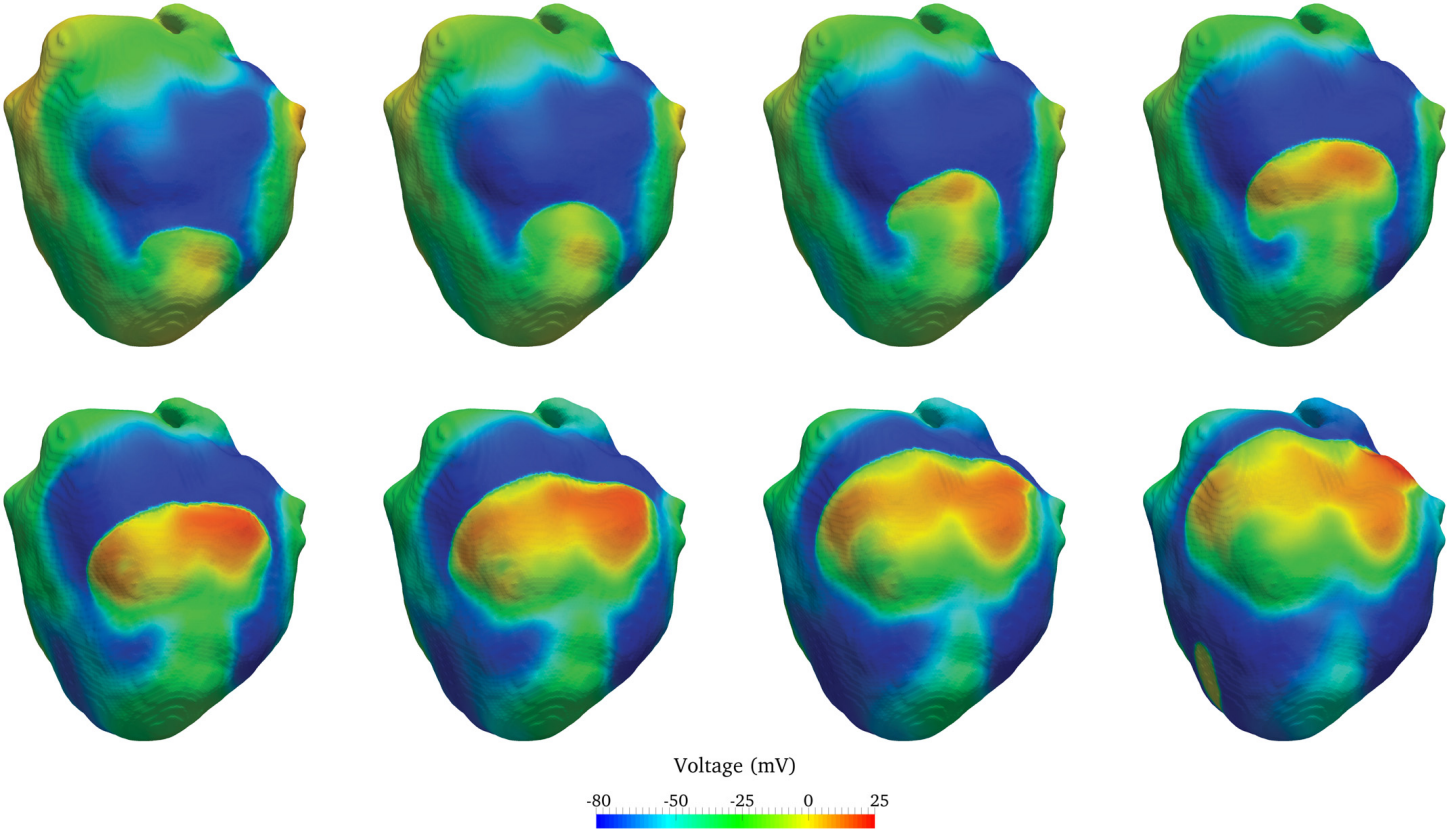
Following the initiation of VF, wavebreak is shown in a time-lapse series of images (left to right, top to bottom) in 10ms intervals. Images show a curved wave of activation reentering resting tissue causing wavebreak. Images start at 1450ms. See also the corresponding ECG in Fig 12 and S1 Movie.

**Fig 12:**
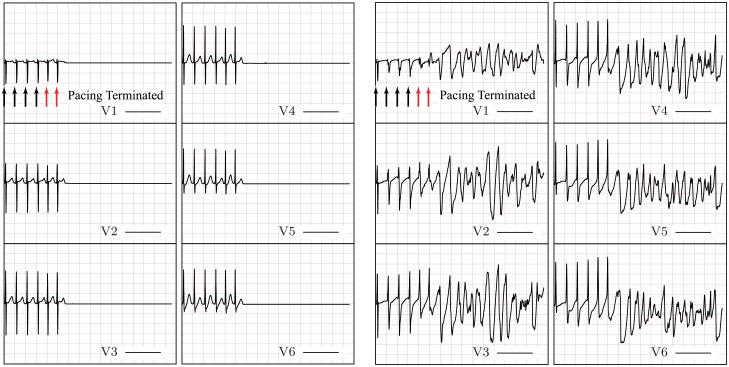
Normal (left) and failing (right) ECG due to four beats at PCL = 200ms (black arrows) followed by two stimuli at PCL = 180ms (red arrows). This rapid pacing protocol causes VF in the failing—but not the normal—biventricular heart model.

The simulations presented in the “T wave and QRS alternans” section show that rapid pacing in a failing heart leads to marked T-wave alternans and subsequently irregular wave propagation at PCL = 200ms. In this setting, can rapid pacing combined with pacing acceleration lead to ventricular fibrillation? As discussed earlier, pacing at 200ms produced irregular T-waves and regional conduction block. However, wavebreak and wave reentry were not observed and every cell returned to its resting state after pacing was terminated. We performed the same test at 180ms and also in this case, the same abnormalities (T-wave alternans, QRS alternans, and conduction block) were observed in the ECG but the heart became electrically silent once pacing was terminated. However, a train of stimuli at 200ms followed by two premature stimuli at 180ms led to wavebreak, reentry, and subsequently sustained chaotic wave propagation, i.e., VF (Fig 11). The same pacing protocol does not lead to VF in the normal heart (Fig 12). The mechanism and onset of VF are characterized by the following key events, which can be observed in the voltage propagation video (S1 Movie):

1. A first beat produces a normal activation sequence and corresponding QRS wave.
2. A region of conduction block appears near the base of the heart in every subsequent beat with pacing interval equal to 200ms. This region grows slightly during every additional beat.
3. The wave of activation circumvents the refractory basal region during the first premature beat at 180ms and tries unsuccessfully to reenter and propagate in already repolarized myocardium.
4. Although the first premature beat at 180ms extinguishes itself, the wave of activation due to a second premature beat goes around the temporarily refractory myocardium, successfully reenters in a mid repolarized region, and finally breaks up leading to VF.
5. Pacing through the Purkinje is stopped at this point and chaotic wave propagation is sustained.

In this case, functionally temporary refractory tissue has created a pathway for reentry, wavebreak, and sustained VF.

**Fig 13:**
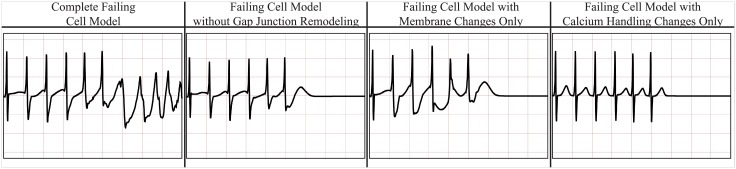
ECG traces for representative lead V5 due to rapid pacing (PCL = 200ms) followed by two accelerated beats (PCL = 180 ms). From left to right: complete failing heart model, heart model without the effect of Connexin 43 alteration, heart model with membrane changes only at the cell level, heart model with calcium handling changes only at the cell level.

In order to determine if all changes considered here to model congestive heart failure were necessary to induce VF, we repeated the rapid pacing simulations with two accelerated beats for three additional models: 1) a model with only the membrane changes at the cell level; 2) a model with only modified calcium handling at the cell level; and 3) a model with no Connexin 43 alteration, i.e., normal conduction velocity. The results for a representative ECG lead are reported in Fig 13 (complete six-lead ECGs are provided in the supporting material) showing that wave break is not sustained in any of the models that contain partial changes. That is, all the changes reported in the foregoing are necessary to significantly increase the susceptibility of the failing heart to VF in our study.

As example of potential applications of our model to study drug therapies targeting heart failure, we consider the effect of SERCA upregulation [41]. In the Mahajan et al. [22] cell model, the SERCA pump is controlled by the equation

$$J_{\text{up}}=\frac{v_{\text{up}}c_{i}^{2}}{c_{i}^{2}+c_{\text{up}}^{2}}$$

In simulating SERCA upregulation, we increase *v*_up_ from 0.27 *μ*M/ms (the value in the failing myocyte) to 0.335 *μ*M/ms, which is the average of the normal (0.4 *μ*M/ms) and failing (0.27 *μ*M/ms) cell model values. Subsequently, we apply the same pacing protocol used to generate VF in the failing heart and observe that the heart becomes electrically silent after pacing is terminated. This suggests that the upregulation of SERCA stops the onset of VF under the proposed mechanism using the protocol and particular pacing described above.

## Discussion

The formulation and validation of the heart failure model presented starts at the single cell level, is investigated at the one dimensional level, and culminates with a geometrically and microstructurally accurate biventricular model. In the following, we highlight the advantages and limitations of the presented approach and discuss our findings. Mirroring the formulation of our model, we examine the single cell model, the one dimensional simulations, and the results obtained at the biventricular level.

### The single myocyte model

In this work, we began by highlighting the ion-channels changes reported by several authors in the literature. We recognize that the reported values characteristic of heart failure (e.g., the peak ion conductances in heart failure) are not unique. Different heart failure patients present different combinations of abnormal ion channels. Moreover, values are reported in the literature only regarding the main peak ionic conductances but, since the implementation of the calcium handling is highly model dependent, no specific values are reported in the literature regarding the strength of calcium reuptake and release for the Mahajan et. al. cell model [22]. In this study, we first modify the membrane ion channels according to the literature and subsequently calibrated the calcium handling changes in order to obtain the calcium transient typical of heart failure. As is true regarding the changes to the membrane ion channels, the changes to calcium handling are also not unique, and separate combinations may lead to similar calcium transients [42].

Although the formulation of our model is not unique—as the myocytes of different failing hearts are not the same—we need to validate our model and reproduce the electrophysiology characteristic of failing myocardial cells. With this aim we have computed action potentials, calcium transients, sodium transients and restitution curves with our cell model in all nine apex-to-base and transmural regions. All these major features agree with the expected electrophysiology of a failing myocyte: longer APD, lower, slower and longer calcium transient, elevated sodium transient, and early alternans onset. Therefore, our cell model represents key phenomena seen in a failing myocyte.

A potential limitation in our cell model regards the limited slope increase of the restitution curve. Indeed, although the onset of alternans occurs early in the failing myocytes, the slope of the restitution curve increased only slightly. We attribute the cause of this potential limitation to the calcium handling formulation. This aspect of the cell model was conceived to well represent rapid pacing and calcium driven alternans in a normal myocyte but further modifications may be needed to best represent the electrophysiology of a failing myocyte. In order to improve this aspect of the single cell model, more experimental data are essential since the restitution curves presented in the heart failure literature show, at times, different features. For example, Glukhov et al. show [43] (slightly) flatter restitution curves in failing human hearts than in normal hearts. On the contrary, Watanabe et al.[44] report steeper restitution curves in the apical myocardium of failing canine hearts. Differences may be due to the specific experimental preparations and protocols or different types of heart failure and a greater understanding of the experimental data would strongly support a more accurate single cell model.

A second limitation of our failing cell model consists in the absence of a late sodium current that instead is replaced by a leak sodium current. This produced the desired effect of having an elevated intracellular sodium in failing cells, but evaluation of a more careful model is warranted.

Finally, recent studies have focused on the importance of SK channel expression [45], and early afterdepolarizations (EADs) [46] and delayed afterdepolarizations (DADs) [15] in heart failure, and on their pro-arrhythmic effect. Currently, these studies are complementary to the one presented here and each aims at understanding the effect of a subset of alterations characterizing heart failure. A future natural extension of the proposed model will incorporate the single cell changes responsible for SK channel expression and EAD/DADs.

### One dimensional simulations

One dimensional simulations are instrumental in understanding the electrophysiology of a group of connected myocytes and the mechanisms leading to ventricular fibrillation in the biventricular model. We observe the early onset of spatially discordant alternans in the one dimensional cable of failing myocytes, but not in the cable made of normal myocytes. Since discordant alternans appears in the homogeneous cable (as well as in the transmural and apex-to-base cables), it is not triggered by the presence of heterogeneities or boundaries between different cell types. Rather the observed discordant alternans is due to the failing cell electrophysiology and, in the examples presented here, is calcium driven. In fact, as shown in failing cell model cable simulations at PCL equal to 250ms (Figs 4, 5 and 6), the DI is approximately 80ms and, correspondingly, the DI/APD restitution curve is fairly flat (slope less than 0.4). This slope will not lead to a voltage driven alternans. On the contrary, calcium transients are significantly longer in the failing myocytes, and subsequent pacing takes place before full calcium recovery from the previous beat has occurred. This is therefore a calcium driven alternans.

At *PCL* ≈ 200ms, discordant alternans is replaced by 2:1 conduction block in both the transmural and apex-to-base failing cables. As discussed in the following, regional blocking plays a fundamental role in initiating the wave break leading to VF in this model.

### Biventricular heart simulations

Linking discordant and concordant APD alternans to a clinical tool like the ECG can provide insights into the detection and diagnosis of heart failure. The delta APD maps corresponding to the failing heart model show the presence of spatially discordant alternans at PCL = 250ms. At the same PCL, the ECG shows marked T-wave alternans with T-wave inversion (Fig 10—failing cell model at PCL = 250ms and Fig 9). A similar situation featuring slightly discordant APD alternans and T-wave alternans (although more modest and without T-wave inversion) is also seen under rapid conditions in the normal heart model (Fig 10—normal cell model at PCL = 250ms and Fig 9). Moreover, in the normal heart, a more marked T-wave alternans corresponds to a larger discordant APD alternans (Fig 10—normal cell model at PCL = 200ms and Fig 9). This suggests that spatially discordant alternans creates marked T-wave alternans in both the normal and, more significantly, in the failing heart model.

As the pacing cycle length is further decreased (PCL = 225 ms), we observe moderate QRS alternans in the ECG obtained using the failing heart model. The amplitude of the QRS wave in subsequent beats is affected by 2:1 blocking of small regions of the myocardium. Indeed, if certain regions of the myocardium are not being activated at every beat, the magnitude of the voltage wave moving past an ECG lead is lower, and consequently the QRS wave for that beat will show a lower amplitude. Finally, as the pacing cycle length is decreased to 200ms, large regional 2:1 blocking occurs at the base of the heart, and this produces the substrate for initiation of wave break and ventricular fibrillation by pacing acceleration.

Linking together the remarks described above at different pacing cycle lengths, we observe that alternans in the T-waves, and subsequently QRS-waves, were precursors to VF induced by pacing acceleration, and may serve as a model to characterize the risk of arrhythmia in patients. We note that Pastore et al. [47] observed similar features in experiments performed on guinea pig hearts at rapid pacing (e.g., Figs. 6 and 8 in [47]). T-wave alternans occurred first, followed by QRS alternans, blocking and finally VF. Similar to Pastore et al., we notice that at rapid pacing, discordant alternans, not concordant alternans, leads to arrhythmia and VF.

There exist several mechanisms to induce VF. Previously, this group has demonstrated the onset of VF using an S1–S2 stimulus protocol in a healthy heart [7]. Cao et al. [48] have shown another mechanism, in which rapid pacing alone, in the presence of non-trivial CV restitution and steep APD restitution, produces spatially discordant alternans leading to wavebreak and VF. However, this is not the mechanism responsible for VF in the current work, since rapid pacing of the failing Mahajan [22] cell model reported here does not steepen the APD restitution curve significantly. Complementing the work of Cao et. al. [48], we aim to explain a new VF mechanism in which dynamic instabilities are triggered by regions of functionally refractory tissue, due to: (1) rapid pacing (four beats at 200ms followed by two beats at 180ms); (2) heart failure cell changes; and (3) apex-to-base APD gradient. The failing cell model shows a longer APD throughout the myocardium and, as a result of the gradients, the APD is further prolonged in the basal region. During subsequent beats, the basal myocardium requires the longest time to repolarize and, at rapid pacing, temporary refractory tissue is present in subsequent beats. As a consequence, the wave of activation during the beats at 180ms encounters a zone of functionally temporarily refractory tissue, circumvents it, and finally breaks and reenters in the mid and basal region once the resting state has been reached. Regions of long and short APDs cause nonuniform wave propagation, which degenerates into wave reentry and sustained chaos due to the high pacing rate and to the lower conduction velocity. A prolonged APD in failing hearts may be a compensatory mechanism with the aim of increasing the time of contraction and offsetting reduced contractile force due to the reduced calcium transient amplitude. Our model suggests that this prolongation in action potential and calcium transient increases the susceptibility to dynamic instabilities under rapid and accelerated heart rhythm.

Several different factors must align to form a favorable substrate to induce VF. In our model, these different factors include changes to the membrane ion currents in the cell model, changes to the cell model calcium handling, and reduction of diffusion anisotropy and magnitude. All these conditions were necessary to induce VF using the proposed rapid pacing protocol, which, on the contrary, was unable to induce VF in the normal heart model or in a failing heart model including only some of these changes. Calcium and membrane changes were essential to induce alternans, and a lower more isotropic diffusion in practice “enlarged” the heart, making it more susceptible to sustained dynamic instabilities.

In this work we have focused on studying the electrophysiology of a failing heart in a mechanically static model and normal anatomy. This allowed us to decouple the pathological electrophysiology from the pathological mechanics and anatomical remodeling due to heart failure. This strategy enabled us to: 1) clearly distinguish the effects of a failing myocyte electrophysiology on ECG, activation maps, and wave of activation; 2) compare directly the new results with our previous work in a healthy heart [7]; and 3) uncover a new EP mechanism that may trigger VF due to rapid heart rates. However, we want to underline that heart failure is a combination of both mechanical and EP changes, and our current model does not include this complex coupling. In adopting this simplification we have not considered, for example, stretch activated channels and increased wall thickness to compensate for decreased contractile forces. Several groups have shown that mechanical changes can cause fluctuations in APDs and even lead to arrhythmias [49]. Therefore, in future work we aim to complement the model proposed here with a mechanical model of contraction and anatomical remodeling to study the risk of ventricular arrhythmia and VF in a fully coupled electromechanical model.

An additional improvement consists in modeling explicitly the presence of fibroblasts following, for example, the work proposed by [9, 50]. Fibroblasts have a higher resting potential with respect to myocytes. Therefore, a large enough group of fibroblasts may activate neighboring myocytes and trigger an ectopic beat that may degenerate in wavebreak and reentry.

#### Clinical implications

The presented model is well suited to investigate the effect of therapies targeting selected ion channels or tissue diffusion, e.g., therapies targeting diffuse fibrosis. As a preliminary example, we have simulated SERCA upregulation and shown that VF is not triggered under the proposed pacing protocol and mechanism. More importantly, the presented model provides a tool to investigate the risk of ventricular fibrillation in patients suffering from heart failure. Defining a classification based on a reliable VF risk factor would be very useful, for instance, in guiding the selection of heart failure patients in need of a ventricular defibrillator. In the current work we have shown that, before the onset of wavebreak and VF, marked T-wave and mild QRS alternans appear in the ECG as the heart rhythm increases in the HF model. These ECG irregularities may become clinical markers to determine the VF risk in HF patients.

### Conclusions

We have constructed a multiscale model to study the electrophysiology of heart failure. We have first validated our model of failing cell electrophysiology against many experimental data reported in the literature. Subsequently, through the monodomain reaction-diffusion equation, we have coupled the single cell electrophysiology to the electrophysiology of anatomically accurate rabbit ventricles. Using the finite element method we have studied how changes at the single cell electrophysiology affect the voltage wave propagation at the tissue and full biventricular level, and the resulting ECG.

This model led to the discovery of a novel mechanism to initiate and sustain VF based on prolonged temporary refractoriness in the heart basal region. The increased basal repolarization time is due to heterogenous APD lengthening, a characteristic feature of HF. We have also shown that changes at the membrane, calcium handling, and tissue levels in cell EP are all responsible and necessary to initiate VF with our protocol and mechanism. Before the onset of wavebreak and VF, T-wave and mild QRS alternans are present in the ECG. Similar results are confirmed experimentally by the work of Pastore et al. [47]. In addition, we show in our model that T-wave alternans is linked to spatially discordant alternans.

We conclude by underlining that additional ion channels or mechanisms may be added to our cell model in a straightforward and modular way. These additional features may both improve the model of the failing myocyte and add ion channels relevant to a particular therapy or a specific form of heart failure.

## Supporting Information

S1 Fig Comparison between healthy and failing myocyte models. Action potentials and calcium transients in nine transmural and apex-to-base regions: (a) Epi-Apex cell region, (b) Epi-Mid cell region, (c) Epi-Base cell region, (d) M-Apex cell region, (e) M-Mid cell region, (f) M-Base cell region, (g) Endo-Apex cell region, (h) Endo-Mid cell region, and (i) Endo-Base cell region.
(EPS)

S2 Fig Uncertainty quantification of action potential duration. Range of action potentials obtained by modifying every cell parameter value by ±10% in each of the nine cell regions: (a) Epi-Apex cell region, (b) Epi-Mid cell region, (c) Epi-Base cell region, (d) M-Apex cell region, (e) M-Mid cell region, (f) M-Base cell region, (g) Endo-Apex cell region, (h) Endo-Mid cell region, and (i) Endo-Base cell region.
(EPS)

S3 Fig Uncertainty quantification of calcium transient. Range of calcium transients obtained by modifying every cell parameter value by ±10% in each of the nine cell regions: (a) Epi-Apex cell region, (b) Epi-Mid cell region, (c) Epi-Base cell region, (d) M-Apex cell region, (e) M-Mid cell region, (f) M-Base cell region, (g) Endo-Apex cell region, (h) Endo-Mid cell region, and (i) Endo-Base cell region.
(EPS)

S4 Fig Sodium transients. Comparison between healthy and failing myocyte models: sodium transients in nine transmural and apex-to-base regions: (a) Epi-Apex cell region, (b) Epi-Mid cell region, (c) Epi-Base cell region, (d) M-Apex cell region, (e) M-Mid cell region, (f) M-Base cell region, (g) Endo-Apex cell region, (h) Endo-Mid cell region, and (i) Endo-Base cell region.
(EPS)

S5 Fig Restitution curves. Dynamic restitution curves obtained using failing and normal myocyte models in nine transmural and apex-to-base regions: (a) Epi-Apex cell region, (b) Epi-Mid cell region, (c) Epi-Base cell region, (d) M-Apex cell region, (e) M-Mid cell region, (f) M-Base cell region, (g) Endo-Apex cell region, (h) Endo-Mid cell region, and (i) Endo-Base cell region.
(EPS)

S6 Fig Normal cell model ECGs. ECGs obtained using the normal biventricular heart model at (a) PCL = 300 ms, (b) PCL = 250 ms, (c) PCL = 225 ms, and (d) PCL = 200 ms.
(EPS)

S7 Fig Failing cell model ECGs. ECGs obtained using the failing biventricular heart model at (a) PCL = 300 ms, (b) PCL = 250 ms, (c) PCL = 225 ms, and (d) PCL = 200 ms.
(EPS)

S8 Fig ECGs with selective cell model changes. ECGs obtained using the failing biventricular heart model at PCL = 200ms for four beats followed by two beats at PCL = 180ms. Wave break and chaotic wave propagation are sustained only in the model containing both membrane and calcium handling cell changes, and slower conduction due to the effect of Cx43 downregulation—Fig 12. The heart becomes electrically silent once pacing is stopped and chaotic wave propagation is not observed when: (a) only membrane current changes are included in the model; (b) only calcium handling changes are included in the model; and (c) membrane current and calcium handling changes are included in the model but conduction values are held normal.
(EPS)

S9 Fig PMJ blocking and retrograde activation. In all three figures, (●) shows the PMJs that remain electrically silent throughout a full beat (PCL = 200ms) in the failing heart model. (a) shows a timepoint where there is conduction block at the Purkinje junction indicated by (→). (b) and (c) show a later timepoint during which PMJs near (■) have retrogradely activated.
(TIF)

S1 Movie VF under rapid pacing. The rapid pacing protocol (four beats at PCL = 200ms followed by two beats at PCL = 180ms) causes VF in the failing biventricular heart model.
(MP4)

## References

[1] Nattel S, Maguy A, Le Bouter S, Yeh YH. Arrhythmogenic ion-channel remodeling in the heart: heart failure, myocardial infarction, and atrial fibrillation. Physiological Reviews. 2007;87(2):425–456. 10.1152/physrev.00014.2006 17429037

[2] Kääb S, Nuss HB, Chiamvimonvat N, O’Rourke B, Pak PH, Kass DA, et al Ionic mechanism of action potential prolongation in ventricular myocytes from dogs with pacing-induced heart failure. Circulation Research. 1996;78(2):262–273. 10.1161/01.RES.78.2.262 8575070

[3] Hoeker GS, Katra RP, Wilson LD, Plummer BN, Laurita KR. Spontaneous calcium release in tissue from the failing canine heart. American Journal of Physiology-Heart and Circulatory Physiology. 2009;297(4):H1235–H1242. 10.1152/ajpheart.01320.2008 19648256 PMC2770773

[4] Qu Z, Garfinkel A, Chen PS, Weiss JN. Mechanisms of discordant alternans and induction of reentry in simulated cardiac tissue. Circulation. 2000;102(14):1664–1670. 10.1161/01.CIR.102.14.1664 11015345

[5] Jongsma HJ, Wilders R. Gap junctions in cardiovascular disease. Circulation research. 2000;86(12):1193–1197. 10.1161/01.RES.86.12.1193 10864907

[6] Keener J, Sneyd J. Mathematical Physiology: I: Cellular Physiology. vol. 1 Springer; 2010.

[7] Krishnamoorthi S, Perotti LE, Borgstrom NP, Ajijola OA, Frid A, Ponnaluri AV, et al Simulation Methods and Validation Criteria for Modeling Cardiac Ventricular Electrophysiology. PLoS ONE. 2014;9(12):e114494 10.1371/journal.pone.0114494 25493967 PMC4262432

[8] Gomez JF, Cardona K, Romero L, Ferrero JM Jr, Trenor B. Electrophysiological and structural remodeling in heart failure modulate arrhythmogenesis. 1D simulation study. PloS one. 2014;9(9):e106602 10.1371/journal.pone.0106602 25191998 PMC4156355

[9] Gomez JF, Cardona K, Martinez L, Saiz J, Trenor B. Electrophysiological and structural remodeling in heart failure modulate arrhythmogenesis. 2D simulation study. PloS one. 2014;9(7):e103273 10.1371/journal.pone.0103273 25054335 PMC4108391

[10] Walmsley J, Rodriguez JF, Mirams GR, Burrage K, Efimov IR, Rodriguez B. mRNA expression levels in failing human hearts predict cellular electrophysiological remodeling: a population-based simulation study. PloS one. 2013;8(2):e56359 10.1371/journal.pone.0056359 23437117 PMC3577832

[11] Moreno JD, Zhu ZI, Yang PC, Bankston JR, Jeng MT, Kang C, et al A computational model to predict the effects of class I anti-arrhythmic drugs on ventricular rhythms. Science translational medicine. 2011;3(98):98ra83–98ra83. 10.1126/scitranslmed.3002588 21885405 PMC3328405

[12] Gomez JF, Cardona K, Trenor B. Lessons learned from multi-scale modeling of the failing heart. Journal of molecular and cellular cardiology. 2015;89:146–159. 10.1016/j.yjmcc.2015.10.016 26476237

[13] Coronel R, Wilders R, Verkerk AO, Wiegerinck RF, Benoist D, Bernus O. Electrophysiological changes in heart failure and their implications for arrhythmogenesis. Biochimica et Biophysica Acta (BBA)-Molecular Basis of Disease. 2013;1832(12):2432–2441. 10.1016/j.bbadis.2013.04.002 23579069

[14] Pogwizd SM, Schlotthauer K, Li L, Yuan W, Bers DM. Arrhythmogenesis and Contractile Dysfunction in Heart Failure Roles of Sodium-Calcium Exchange, Inward Rectifier Potassium Current, and Residual *β*-Adrenergic Responsiveness. Circulation Research. 2001;88(11):1159–1167. 10.1161/hh1101.091193 11397782

[15] Shannon TR, Wang F, Bers DM. Regulation of cardiac sarcoplasmic reticulum Ca release by luminal [Ca] and altered gating assessed with a mathematical model. Biophysical Journal. 2005;89(6):4096–4110. 10.1529/biophysj.105.068734 16169970 PMC1366975

[16] Rose J, Armoundas AA, Tian Y, DiSilvestre D, Burysek M, Halperin V, et al Molecular correlates of altered expression of potassium currents in failing rabbit myocardium. American Journal of Physiology-Heart and Circulatory Physiology. 2005;288(5):H2077–H2087. 10.1152/ajpheart.00526.2003 15637125 PMC2711868

[17] Tsuji Y, Opthof T, Kamiya K, Yasui K, Liu W, Lu Z, et al Pacing-induced heart failure causes a reduction of delayed rectifier potassium currents along with decreases in calcium and transient outward currents in rabbit ventricle. Cardiovascular Research. 2000;48(2):300–309. 10.1016/S0008-6363(00)00180-2 11054476

[18] Pogwizd SM, Bers DM. Na/Ca Exchange in Heart Failure. Annals of the New York Academy of Sciences. 2002;976(1):454–465. 10.1111/j.1749-6632.2002.tb04775.x 12502595

[19] Winslow RL, Rice J, Jafri S, Marban E, O’Rourke B. Mechanisms of altered excitation-contraction coupling in canine tachycardia-induced heart failure, II Model studies. Circulation Research. 1999;84(5):571–586. 10.1161/01.RES.84.5.571 10082479

[20] Noble D, Noble P. Late sodium current in the pathophysiology of cardiovascular disease: consequences of sodium–calcium overload. Heart. 2006;92(suppl 4):iv1–iv5. 10.1136/hrt.2005.078782 16775091 PMC1861316

[21] Despa S, Islam MA, Weber CR, Pogwizd SM, Bers DM. Intracellular Na+ concentration is elevated in heart failure but Na/K pump function is unchanged. Circulation. 2002;105(21):2543–2548. 10.1161/01.CIR.0000016701.85760.97 12034663

[22] Mahajan A, Shiferaw Y, Sato D, Baher A, Olcese R, Xie LH, et al A rabbit ventricular action potential model replicating cardiac dynamics at rapid heart rates. Biophysical Journal. 2008;94(2):392–410. 10.1529/biophysj.106.98160 18160660 PMC2157228

[23] Ai X, Pogwizd SM. Connexin 43 downregulation and dephosphorylation in nonischemic heart failure is associated with enhanced colocalized protein phosphatase type 2A. Circulation Research. 2005;96(1):54–63. 10.1161/01.RES.0000152325.07495.5a 15576650

[24] Akar FG, Spragg DD, Tunin RS, Kass DA, Tomaselli GF. Mechanisms underlying conduction slowing and arrhythmogenesis in nonischemic dilated cardiomyopathy. Circulation Research. 2004;95(7):717–725. 10.1161/01.RES.0000144125.61927.1c 15345654

[25] Kitamura H, Ohnishi Y, Yoshida A, Okajima K, Azumi H, Ishida A, et al Heterogeneous loss of connexin43 protein in nonischemic dilated cardiomyopathy with ventricular tachycardia. Journal of cardiovascular electrophysiology. 2002;13(9):865–870. 10.1046/j.1540-8167.2002.00865.x 12380923

[26] Dupont E, Matsushita T, Kaba RA, Vozzi C, Coppen SR, Khan N, et al Altered connexin expression in human congestive heart failure. Journal of molecular and cellular cardiology. 2001;33(2):359–371. 10.1006/jmcc.2000.1308 11162139

[27] Hooks DA, Trew ML, Caldwell BJ, Sands GB, LeGrice IJ, Smaill BH. Laminar arrangement of ventricular myocytes influences electrical behavior of the heart. Circulation Research. 2007;101(10):e103–e112. 10.1161/CIRCRESAHA.107.161075 17947797

[28] Perotti L, Krishnamoorthi S, Borgstrom N, Ennis D, Klug W. Regional segmentation of ventricular models to achieve repolarization dispersion in cardiac electrophysiology modeling. International journal for numerical methods in biomedical engineering. 2015;31(8). 10.1002/cnm.2718 25845576 PMC4519348

[29] Holzem KM, Efimov IR. Arrhythmogenic remodelling of activation and repolarization in the failing human heart. Europace. 2012;14(suppl 5):v50–v57. 10.1093/europace/eus275 23104915 PMC3697802

[30] Elshrif MM, Shi P, Cherry EM. Representing Variability and Transmural Differences in a Model of Human Heart Failure. Biomedical and Health Informatics, IEEE Journal of. 2015;19(4):1308–1320. 10.1109/JBHI.2015.2442833 26068919

[31] Han W, Chartier D, Li D, Nattel S. Ionic remodeling of cardiac Purkinje cells by congestive heart failure. Circulation. 2001;104(17):2095–2100. 10.1161/hc4201.097134 11673352

[32] Li GR, Lau CP, Ducharme A, Tardif JC, Nattel S. Transmural action potential and ionic current remodeling in ventricles of failing canine hearts. American Journal of Physiology-Heart and Circulatory Physiology. 2002;283(3):H1031–H1041. 10.1152/ajpheart.00105.2002 12181133

[33] Qu Z, Garfinkel A. An advanced algorithm for solving partial differential equation in cardiac conduction. IEEE Transactions on Biomedical Engineering. 1999;46(9):1166–1168. 10.1109/10.784149 10493080

[34] Krishnamoorthi S, Sarkar M, Klug WS. Numerical quadrature and operator splitting in finite element methods for cardiac electrophysiology. International journal for numerical methods in biomedical engineering. 2013;29(11):1243–1266. 10.1002/cnm.2573 23873868 PMC4519349

[35] Vermeulen JT, Tan HL, Rademaker H, Schumacher CA, Loh P, Opthof T, et al Electrophysiologic and extracellular ionic changes during acute ischemia in failing and normal rabbit myocardium. Journal of molecular and cellular cardiology. 1996;28(1):123–131. 10.1006/jmcc.1996.0012 8745220

[36] Kleber AG, Janse MJ, Wilms-Schopmann F, Wilde A, Coronel R. Changes in conduction velocity during acute ischemia in ventricular myocardium of the isolated porcine heart. Circulation. 1986;73(1):189–198. 10.1161/01.CIR.73.1.189 3940667

[37] Akar FG, Nass RD, Hahn S, Cingolani E, Shah M, Hesketh GG, et al Dynamic changes in conduction velocity and gap junction properties during development of pacing-induced heart failure. American Journal of Physiology-Heart and Circulatory Physiology. 2007;293(2):H1223–H1230. 10.1152/ajpheart.00079.2007 17434978

[38] Corrias A, Giles W, Rodriguez B. Ionic mechanisms of electrophysiological properties and repolarization abnormalities in rabbit Purkinje fibers. American Journal of Physiology-Heart and Circulatory Physiology. 2011;300(5):H1806–H1813. 10.1152/ajpheart.01170.2010 21335469

[39] Panfilov AV, Holden AV, et al Computational biology of the heart. Wiley; 1997.

[40] Niederer SA, Kerfoot E, Benson AP, Bernabeu MO, Bernus O, Bradley C, et al Verification of cardiac tissue electrophysiology simulators using an N-version benchmark. Philosophical Transactions of the Royal Society of London A: Mathematical, Physical and Engineering Sciences. 2011;369(1954):4331–4351. 10.1098/rsta.2011.0139 PMC3263775 21969679

[41] Bassani JW, Bassani RA. SERCA upregulation: breaking the positive feedback in heart failure? Cardiovascular research. 2005;67(4):581–582. 10.1016/j.cardiores.2005.06.019 16018992

[42] Weiss JN, Karma A, MacLellan WR, Deng M, Rau CD, Rees CM, et al “Good enough solutions” and the genetics of complex diseases. Circulation Research. 2012;111(4):493–504. 10.1161/CIRCRESAHA.112.269084 22859671 PMC3428228

[43] Glukhov AV, Fedorov VV, Lou Q, Ravikumar VK, Kalish PW, Schuessler RB, et al Transmural dispersion of repolarization in failing and nonfailing human ventricle. Circulation Research. 2010;106(5):981–991. 10.1161/CIRCRESAHA.109.204891 20093630 PMC2842469

[44] Watanabe T, Yamaki M, Yamauchi S, Minamihaba O, Miyashita T, Kubota I, et al Regional prolongation of ARI and altered restitution properties cause ventricular arrhythmia in heart failure. American Journal of Physiology-Heart and Circulatory Physiology. 2002;282(1):H212–H218. 11748065 10.1152/ajpheart.2002.282.1.H212

[45] Hsieh YC, Chang PC, Hsueh CH, Lee YS, Shen C, Weiss JN, et al Apamin sensitive potassium current modulates action potential duration restitution and arrhythmogenesis of failing rabbit ventricles. Circulation: Arrhythmia and Electrophysiology. 2013;p. CIRCEP–112. 10.1161/CIRCEP.111.000152 PMC3678988 23420832

[46] Passini E, Mincholé A, Coppini R, Cerbai E, Rodriguez B, Severi S, et al Mechanisms of pro-arrhythmic abnormalities in ventricular repolarisation and anti-arrhythmic therapies in human hypertrophic cardiomyopathy. Journal of molecular and cellular cardiology. 2015;. 26385634 10.1016/j.yjmcc.2015.09.003 PMC4915817

[47] Pastore JM, Girouard SD, Laurita KR, Akar FG, Rosenbaum DS. Mechanism linking T-wave alternans to the genesis of cardiac fibrillation. Circulation. 1999;99(10):1385–1394. 10.1161/01.CIR.99.10.1385 10077525

[48] Cao JM, Qu Z, Kim YH, Wu TJ, Garfinkel A, Weiss JN, et al Spatiotemporal heterogeneity in the induction of ventricular fibrillation by rapid pacing importance of cardiac restitution properties. Circulation Research. 1999;84(11):1318–1331. 10.1161/01.RES.84.11.1318 10364570

[49] Franz MR, Burkhoff D, Yue DT, Sagawa K. Mechanically induced action potential changes and arrhythmia in isolated and in situ canine hearts. Cardiovascular research. 1989;23(3):213–223. 10.1093/cvr/23.3.213 2590905

[50] McDowell KS, Arevalo HJ, Maleckar MM, Trayanova NA. Susceptibility to arrhythmia in the infarcted heart depends on myofibroblast density. Biophysical Journal. 2011;101(6):1307–1315. 10.1016/j.bpj.2011.08.009 21943411 PMC3177053

