# Supplementary figures and images for: Electrophysiology of Heart Failure Using a Rabbit Model: From the Failing Myocyte to Ventricular Fibrillation
Source: PLoS Comput Biol. 2016 Jun 23;12(6):e1004968. doi: 10.1371/journal.pcbi.1004968 (PMC4919062; doi:10.1371/journal.pcbi.1004968)

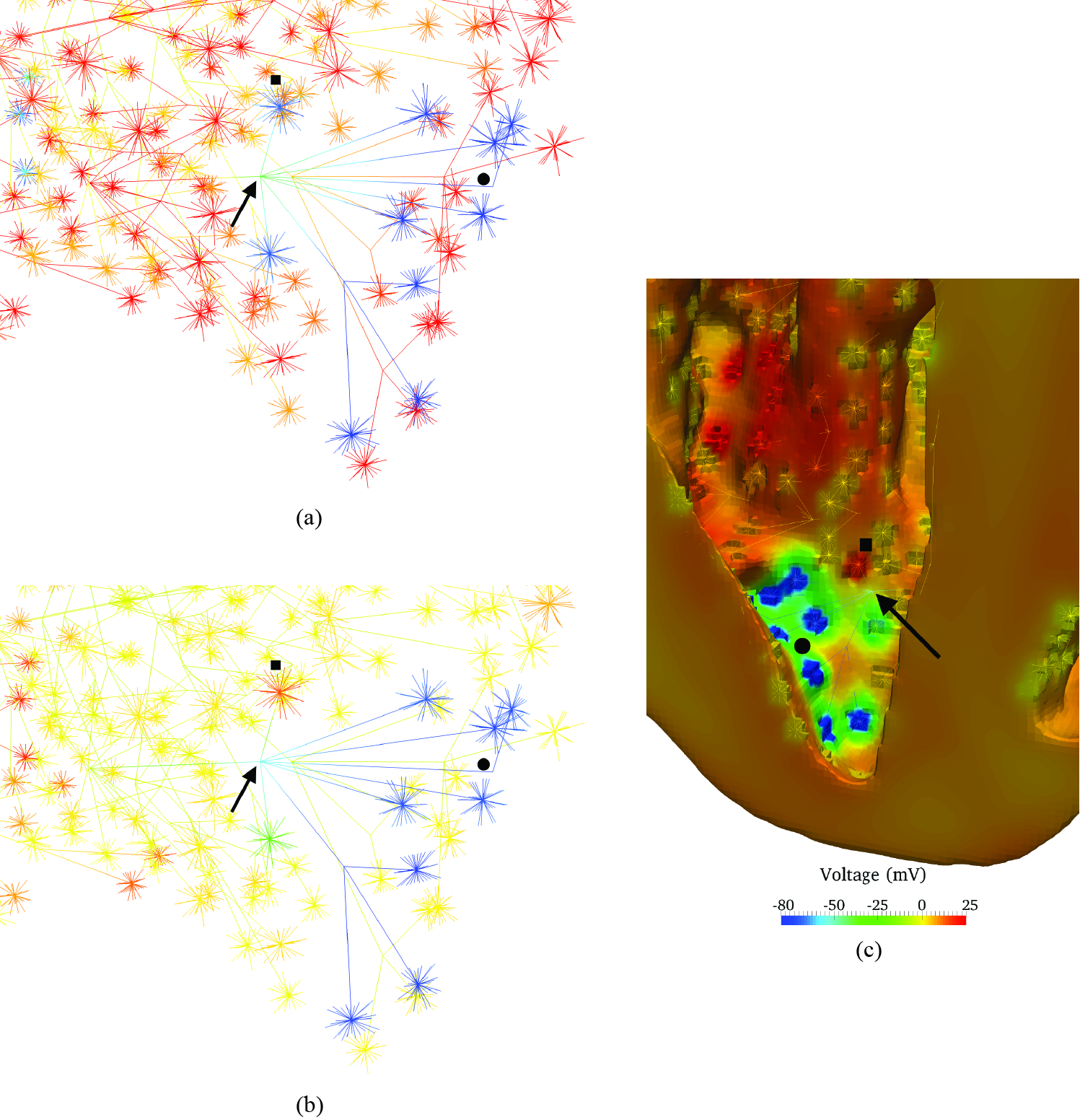

Supplement: S9 Fig — In all three figures, (●) shows the PMJs that remain electrically silent throughout a full beat (PCL = 200ms) in the failing heart model. (a) shows a timepoint where there is conduction block at the Purkinje junction indicated by (→). (b) and (c) show a later timepoint during which PMJs near (■) have retrogradely activated. (TIF) [file pcbi.1004968.s009.tif]
